# Autism spectrum disorder, politics, and the generosity of insurance mandates in the United States

**DOI:** 10.1371/journal.pone.0217064

**Published:** 2019-05-24

**Authors:** Timothy Callaghan, Steven Sylvester

**Affiliations:** 1 Department of Health Policy and Management, Texas A&M University, School of Public Health, College Station, Texas, United States of America; 2 Department of History and Political Science, Utah Valley University, Orem, Utah, United States of America; Chinese Academy of Medical Sciences and Peking Union Medical College, CHINA

## Abstract

The study of Autism Spectrum Disorder (ASD) in the United States has identified a growing prevalence of the disorder across the country, a high economic burden for necessary treatment, and important gaps in insurance for individuals with autism. Confronting these facts, states have moved quickly in recent years to introduce mandates that insurers provide coverage for autism care. This study analyzes these autism insurance mandates and demonstrates that while states have moved swiftly to introduce them, the generosity of the benefits they mandate insurers provide varies dramatically across states. Furthermore, our research finds that controlling for policy need, interest group activity, economic circumstances, the insurance environment, and other factors, the passage of these mandates and differences in their generosity are driven by the ideology of state residents and politicians–with more generous benefits in states with more liberal citizens and increased Democratic control of state government. We conclude by discussing the implications of these findings for the study of health policy, politics, and autism in America.

## Introduction

Autism spectrum disorder (ASD) is a neurodevelopmental condition caused by complex hereditary and environmental factors that can lead to social, communication, and behavioral challenges [1–3]. Since 2000, the Centers for Disease Control and Prevention estimates that the diagnosed prevalence of ASD among children has increased from 1 in 150 to 1 in 68 [3]. This increased rate of diagnosis has driven researchers to investigate both the root causes of ASD as well as the impact of ASD on patients, their families, and the healthcare industry. Scholars have devoted particular attention to the economic burden of ASD [4–7]. Recent research suggests that yearly medical costs and other behavioral treatments for ASD can range from $6,000 to more than $35,000 per child in the first 5 years of life [8–10]. Critically however, many of these costs have not been covered by insurance companies and instead have been shifted to the parents and families of individuals with ASD [11].

This increased prevalence of ASD, and its associated out of pocket costs, has drawn interest from advocacy groups who have put pressure on lawmakers to decrease this cost burden on individuals with ASD and their families [12]. These efforts have centered on pursuing policy changes to mandate insurance coverage of ASD in states across the country [12, 13–14]. In particular, legislative efforts have emphasized the importance of insurance companies providing basic medical services as well as habilitative services like Applied Behavior Analysis (ABA) to individuals with ASD. Research has shown that early habilitative treatments like ABA can improve development; however, without intervention in the form of insurance mandates, most insurance companies refuse to cover even basic ASD care and treatment due to its high cost [9, 15].

Responding to the growing rate of diagnosis and pressure from ASD advocates, state legislatures passed mandates in 46 states from 2001–2017 requiring insurance companies to cover services associated with ASD. However, as we will show, the generosity of these mandates–which we define as a function of both the volume of services required to be provided by private insurers as well as the age of individuals eligible for these services–varies dramatically across states. While some state mandates require benefits designed to ensure adequate medical care for all individuals with ASD, benefits in other states are more limited–cutting off program eligibility before the age of 10 or capping yearly covered medical costs far below what many families spend each year on care.

With these generosity differences determining whether or not large segments of the ASD population in each state are covered by insurance, understanding the determinants of these variations in generosity is an important area of inquiry. This is particularly true given new research suggesting that the presence of–and differences in–autism insurance mandate generosity alter service utilization [12, 14, 16–18]. Most notably, Kennedy-Hendricks et al. 2018 present convincing evidence that age caps on service eligibility–a key component of our benefit generosity index–significantly reduce health service use and ASD-related spending, particularly in the outpatient setting [16]. With these new findings in mind, we analyze why some states are more generous in their regulations surrounding ASD insurance mandates than others. In doing so, our research not only provides crucial information about the development and scope of ASD mandates, but also points to the need for studies of insurance mandates more broadly to account for generosity.

### The current state of autism policy

Scholars have devoted considerable attention to ASD policy in recent years [9, 12, 14, 16–23]. Like many areas of health policy, this research consistently demonstrates the contentious nature of policymaking regarding ASD. As noted by Marmor (2017), ASD policy is “an arena of extraordinary complexity and conflict for those navigating within it,” with “a bewilderingly complex arena of actors” at work [24]. This conflict can be traced to a complex tapestry of public policies designed to provide needed services for individuals with ASD. This includes not just insurance regulations, which are the focus of this research, but also educational programs, long-term care programs, and other key social services [18].

This intricate web of regulations and social services surrounding ASD makes it difficult for lawmakers to come to a consensus on policy solutions in the ASD arena, in large part due to the high cost for these programs. Policy scholars regularly point to contentious ASD policy debates over who should deliver treatment and who should be responsible for payment given the strain that these programs can put on state and local budgets that are often underfunded [25–26].

For example, the federal government regulates certain aspects of ASD therapy in public school special education programs through the Individuals with Disabilities Education Act (IDEA). However, each state has the flexibility to determine program eligibility requirements for children with ASD so long as the states meet the minimum requirements set forth by the federal government [22, 27–28]. This shared governance complicates policy efforts and opens eligibility rules to a variety of sub-national political actors with different strategic interests related to the government provision of social services. Thus, despite federal regulations designed to ensure basic ASD therapies, one child may be eligible for many additional services in one state but may not be eligible for any in another state.

Furthermore, while key aspects of treatment for ASD occur within the school system, many necessary ASD services are not available in this context. For example, in a study examining special education services received by students with ASD from preschool to high school, Wei et al. (2014) found that even as most students receive speech/language therapy through the school system, few schools provide ABA or any other type of behavior management program [29]. This is consistent with previous research that has suggested that public schools have paid little attention to behavior management therapy for ASD, in part due to its high cost [30–32]. The lack of attention to behavior-based therapies in the school system is also partly attributable to the focus of educational institutions in fulfilling their core mission of education, which behavioral management could be argued to fall outside of.

Given these school based limits, individuals with ASD and their families often seek out private medical care to fill the gaps in needed treatments, particularly in the area of habilitative services. These behavioral treatments like ABA vary dramatically in cost based on condition severity and state of residence, but expenses typically fall between $10,000 and $100,000 per year [33–34]. While this cost is borne by insurance companies in some cases, most insurance companies have historically been hesitant to cover behavioral treatment, arguing that these medical services are experimental and not “medically necessary” [9, 33] despite evidence that ABA increases socially acceptable behavior in individuals with ASD [35–36]. This has forced many families to bear this cost themselves and prevented countless others from getting these services at all.

Those impacted by ASD had hoped for relief with the passage of the Paul Wellstone and Pete Domenici Mental Health Parity and Addiction Equity Act (MHPAEA) in 2008 and the Affordable Care Act (ACA) in 2010; however, their impact on forcing insurance companies to provide needed care has been limited. While MHPAEA prevents insurers from treating mental health differently than other medical care and from imposing lifetime dollar limits on mental health services, it does not specify the conditions that must be covered [14, 37]. Given this issue, states have been slow to recognize ASD as a mental health care condition that must comply with MHPAEA [12]. When they have, insurers have fought vociferously to prevent habilitative services from being classified as medically necessary, leading to litigation in many cases [38–40].

Furthermore, while the ACA’s Essential Health Benefits (EHB) require marketplace plans to provide mental health services including behavioral health treatment, their impact on insurers in the provision of ASD has been vague at best [41]. Even as the ACA mandates coverage for mental health, benchmark plans may or may not cover habilitative services designed to help individuals with ASD function in their day-to-day lives [42–43]. Given these issues, individuals impacted by ASD have pushed advocacy groups and, in turn, state legislators to create state mandates for insurance that covers all types of ASD therapy [44–46].

This intense pressure has created two changes critical to our research. First, sensing pressure from their constituents, Autism Speaks and its Office of State Governmental Affairs has prioritized the topic and led a nationwide charge for the enactment of state mandates that insurance companies cover needed ASD services [38]. Second, with activists and interest groups pushing for change, states have moved rapidly to introduce and enact ASD insurance mandates. While there were no ASD insurance mandates as recently as 2000, we show that 46 states had enacted ASD mandates by the end of 2017. It is important to note that the enactment of these mandates predates both MHPAEA and the ACA and should not be considered to be a mere result of the enactment of these federal policies. The first ASD insurance mandate was passed in 2001 and several states had passed mandates by the time MHPAEA was passed in 2008. By the time MHPAEA was fully implemented in private insurance markets and enrollment in the ACA’s health insurance exchanges began in 2013, almost 40 states had passed ASD insurance mandates [39].

Critically, not all state ASD insurance mandates are equally generous. Even as some states have passed mandates that require insurers to provide needed treatments for all residents with ASD, other states have elected to limit eligibility to subsets of individuals with ASD. The decision to mandate insurance coverage for ASD and to set limitations on eligibility has significant policy implications, and yet, research on what drives those state decisions is limited. The purpose of this study is to provide a first ever test of the determinants of insurance mandate generosity to understand both the causes of different ASD generosity levels across the 50 states and the importance of accounting for differences in insurance mandate generosity more broadly.

### Health insurance mandates and their generosity

Our analysis of the generosity of ASD insurance mandates fits within an important literature on insurance mandates within the states. Mandated benefit legislation requires insurers to cover needed health care services for a variety of medical condition and can take several different forms [40, 47–49]. They can require insurers to cover particular conditions or services, require that insurers cover services from certain types of providers, require that coverage is available to subgroups of the population, or do some combination of all three [49].

Typically developed in response to consumer frustration over the limited coverage offered by health insurers, mandates have become an increasingly popular strategy used by states over time [50]. For example, Laugesen et al. (2006) notes that “between 1970 and 1996, there was a 25-fold increase in mandated benefit laws enacted,” a trend which has only intensified in recent years [40]. This is because mandated benefits are an attractive strategy for state legislators–they do not raise taxes or impact government revenues, they satisfy interest group pressure, and have proven to be effective in a variety of settings [47, 51]. The popularity and effectiveness of insurance mandates has been borne out by recent health services research, which has pointed to the importance of mandates related to infertility, maternity length of stay, cancer screening, mental health, and many other topics [14, 47, 52–55].

Additional research has focused on the enactment and utilization of ASD insurance mandates, which are the focus of our study [9, 12, 16, 17]. While ASD mandates are similar to other insurance mandates in their focus on requiring insurers to cover needed health care services, the scope and speed of their adoption has been unusual. In a comparative analysis of 1,471 insurance mandates from 1949–2002, Laugesen et al. (2006) show that only two mandates were adopted by all states and both were federally mandated [40]. Broad ASD benefits are not federally mandated, and yet, 46 states passed ASD mandates from 2001–2017 [38]. This rapid and widespread enactment might be attributable to a variety of factors including intense interest group pressure, federal legislation in MHPAEA and the ACA that have left ASD benefits vague, and a population that the public believes should be insured.

Despite the importance of this research, both on mandates in general and ASD in particular, one key gap in the insurance mandate literature remains–little is known about the causes and consequences of variations in mandate generosity. For example, Rathore et al. (2000) when analyzing mandates for cancer screening services note that “although differences in state coverage mandates were expected, the rationale for variation is unclear” [53]. Similarly, Laugesen et al. (2006) point out that “despite a growing number of mandated benefit laws enacted at the state level, comparative analyses of mandated benefit laws are unusual” [40]. Put simply, differences in insurance mandate generosity are regularly noted but rarely analyzed at either the enactment or utilization phase. With the generosity of each mandate determining which state residents are eligible for these services, understanding the determinants of the generosity of each mandate at enactment represents an important area of inquiry.

Using existing research on mandates and health policy generosity as guides, our research investigates the potential roles of six factors in explaining variations in the generosity of ASD mandates: partisanship, state economic circumstance, legislative professionalism, interest group activity, policy diffusion, and the state insurance environment.

## State determinants of mandate generosity

### Partisanship

Partisanship is the “workhorse” of American politics at the state and national levels and serves as an important starting point in discussions of differences in the generosity of insurance mandates across states [56–57]. Policies related to the role of government in the provision of health care and the regulation of insurance companies can differ dramatically between legislatures controlled by liberal politicians and conservative politicians. As demonstrated by past research on the ACA and other health care policies, these political differences are often the defining difference between state actions, with liberal states more likely to enact generous policies that expand the social welfare state [58–63]. In the case of ASD mandates, whether a state chooses to enact a mandated benefit law that provides benefits to all those who need them or just to subsets of the population could similarly result from differences in ideology at the mass and elite levels.

That said the impact of politics on ASD policy remains unclear. For example, Marmor (2017) notes that “there are few if any studies of autism as an instance of substantial policy and politics,” and there are reasons to believe that traditional ideological patterns may not hold [24]. While we would expect liberal states to be more generous in the provision of mandated benefits based on past behavior in the enactment of health and social policy, prior research by Johnson et al. (2014) on whether or not a state has an ASD mandate instead finds that Republican states are more likely to pass a mandate [9]. In addition, Republican legislators dominate policymaking in many of the earliest adopting states including Indiana, South Carolina, and Texas. Even with these caveats however, with a massive body of literature in health policy pointing to the importance of partisanship, accounting for partisanship in our analysis is critical.

### State economic circumstances

The economic circumstances within each state serve as a potentially potent alternative mechanism beyond politics that could explain differences in the generosity of ASD mandates. Prior research in health policy demonstrates that state economic conditions alter the scope of health policy generosity [57]. Even when states are open to the enactment of policies that will create new fiscal commitments, affluent states can more easily take on new financial burdens and thus, are more likely to enact policies to cover what is needed, as opposed to just what the state budget and state residents can bear [60, 64–65].

Prior research on mandated benefit laws also points to the importance of accounting for economic circumstances. Insurance mandates have been shown to consistently raise the price of insurance coverage in states and expensive mandates have been shown to be adopted and revised by legislatures less often [40, 49]. Economic circumstances could play a particularly important role in the case of ASD mandates. Costs for necessary medical and habilitative services are quite high and with a growing diagnosis rate, legislators in poorer states may be hesitant to institute a mandate that could dramatically increase the cost of insurance over time if diagnosis rates continue to climb.

### Legislative professionalism

The professionalism of the legislature in each state could also help to explain differences in ASD benefit generosity. Enacting complex public policies requires a clear understanding of subject-specific information and legislatures that have the time and resources to develop that knowledge could enact policies that look quite different from policies enacted by citizen legislatures [66]. Supporting this idea, a large body of research has found that differences in state legislative professionalism explain a variety of state policy outcomes [67–70]. In the case of ASD mandates, we might expect to find that professional legislatures have the time and resources to learn about the needs of individuals with ASD and will enact generous benefits to fit those needs. Alternatively, more professional legislatures could be more likely to understand the potential fiscal strain of these mandates on state insurers and enact less generous mandates to work against the concerns of this prominent industry group.

### Interest group activity

Interest groups have also been shown to play an important role in the passage of mandated benefit legislation and are therefore a necessary factor to consider in our analysis of ASD generosity. In particular, prior research demonstrates that in the case of mandated benefit legislation, the voices of groups with a direct stake in the policy are not only listened to but often drive policy debates [40, 50]. This is particularly true in the case of ASD, where advocacy groups and the insurance industry jockey for position.

On one hand, Autism Speaks pursues the enactment of generous mandated benefit legislation as a top priority [13]. To this end, they provide expert testimony, encourage grassroots campaigns, and even hold a yearly summit designed to teach policy entrepreneurs how to navigate the policymaking process to enact them [35]. On the other, insurance companies and their lobbies fight to prevent the enactment of a mandate and short that, to limit eligibility to preserve their bottom lines. Mandell et al. (2016), for example, note that insurance companies resist ASD mandates by arguing that they increase the number of individuals who are diagnosed with ASD and dramatically increase health spending [12]. Critically, with business organizations–in this case the insurance industry–presenting a united front in opposition to these mandates, we would expect their resource advantages to dominate states with a high concentration of health interest groups and limit the generosity of enacted mandates [57, 71–72].

### Policy diffusion

Prior research on the enactment of state policies demonstrates that policy diffusion can also play an important role in the spread of policies across the United States and is therefore an important factor to consider in our analysis of autism insurance mandates. Scholars have shown across a number of policy areas that states are more likely to enact policies after observing the policies enacted in other states–pairing what they learn from other states with information from professional organizations, advocates, and interest groups [73–75]. The potential influence of diffusion to policy decisions like the enactment of generous mandates is perhaps best articulated by Berry and Berry (1990). They note that the likelihood of a state adopting a policy is a function of the internal characteristics to the state (i.e. the ideology of state representatives, interest group activity, and legislative professionalism), as well as factors external to a state, such as the behavior of neighboring states. The role of diffusion in state policymaking has been studied in prominent policy areas including DUI policy, health insurance programs for children, and LGBTQ issues and is important to include here because the behavior of neighboring states could alter a state’s likelihood of adopting a generous ASD insurance mandate [76–78].

### State insurance environment

The insurance environment in each state could also help to explain differences in the generosity of ASD mandates in a variety of ways. First, mandated benefit laws are only applicable to state residents with private insurance that are not part of self-insured firms. This is because the Employee Retirement Income Security Act (ERISA) exempts self-insured plans from state insurance benefit legislation [14, 40]. Therefore, we might expect that states with less self-insured firms, and thus a larger pool of eligible residents, may be more likely to enact generous benefits. Additionally, differences in generosity could be driven by the number of uninsured state residents or by the number of individuals with ASD. If a state has a larger number of uninsured individuals or residents with ASD, they might perceive a higher need for generous benefits to cover these populations and enact state mandates that ensure adequate coverage.

## Materials and methods

To investigate ASD insurance mandates across the states, our study relies on a careful analysis of every ASD insurance mandate passed in the US from 2000–2017. ASD legislation for this study was obtained from the National Conference of State Legislatures’ Autism and Insurance Coverage Dataset and the American Speech Language Hearing Association Insurance Mandate Dataset. This information was further supplemented with information gained by the authors using the legislative tracking website Legiscan. Together, these sources allowed us to create a comprehensive set of every piece of legislation enacted in the US states designed to mandate insurance coverage for ASD.

Our analysis proceeds in two stages. First, we present an updated look at the predictors of enacting any ASD insurance mandate which was last explored in Johnson et al. 2014 –an analysis that presented data through 2012 [9]. That initial analysis relies on a 0/1 indicator measure as its dependent variable where states are scored as zeros until the year in which they first enact an ASD insurance mandate where they are coded as a one. Then, we move on to our primary analysis where we present the results of our study of variations in benefit generosity across insurance mandates. As no clear guidance exists on an appropriate measure of benefit generosity in this context, we adopted a new approach for our generosity dependent variable that is tailored to the population of interest and the way in which these mandates were written. Specifically, we created a coding scheme for benefit generosity that accounts for the presence of an insurance mandate, age restrictions on benefits, spending caps on benefits, and inflation adjustment.

In our measure, the lowest potential score is zero and that was awarded to states that had no ASD insurance coverage mandate. These states do not specify that insurance companies must provide health coverage to individuals with ASD and as such, benefits in these states are limited to the essential health benefits mandated by the ACA. Critically, without a mandate, insurance coverage in these states excludes the ABA and other forms of habilitative treatment needed for proper ASD care.

Next, states were awarded a generosity score of one in our analysis if they successfully passed an ASD insurance coverage mandate, but it was restricted by both age and spending caps. Specifically, a state was awarded a score of one if the benefits were restricted to individuals under 18 and if the legislation set a spending cap for necessary treatment below the median spending cap for all states. States in this category recognized the importance of passing a mandate but elected to limit its access to children and to put stringent caps on spending. For example, Oklahoma’s 2016 mandate requires insurers to provide coverage for those with ASD, but mandates coverage only until age 9 and sets a low annual maximum benefit of $25,000 [79].

Additional points were awarded in our coding scheme if states passed an ASD insurance mandate and were generous in terms of age restrictions OR spending caps (worth 1 point each). In other words, if the state allowed adults to obtain benefits or if their spending cap was above the median spending cap value, they were given a second point. Alternatively, if the state passed a mandate that provided coverage for adults AND that was over the median benefit spending cap then the state was given three points. These categories are exemplified by Arkansas and Kentucky respectively. Arkansas has a generous spending cap of $50,000 annually but only allows individuals under 18 to access it and was scored as a 2. Kentucky on the other hand, has a $50,000 spending cap but allows individuals to access benefits until they turn 21 and was scored as a 3 [79].

Next, a state was given the highest possible generosity score, a four, if the state passed an ASD insurance mandate that had no age restrictions at all and if there is no spending cap written into the legislation. States in this category mandate that insurance companies provide generous benefits to help ensure adequate treatment for individuals with ASD. For example, the mandated benefit law passed by Massachusetts requires insurers in the state to provide coverage for mental disorders including ASD without setting any limits based on age or spending caps [79].

Lastly, as the value of a yearly spending cap erodes over time due to inflation, we feel that states with spending caps that do not account for this in their laws produce legislation that will inherently be less generous over time. For that reason, we subtract a half point from states who pass mandates with yearly spending caps but no adjustment for inflation in the law. Therefore, states with half points (i.e. 1.5 or 2.5) on our generosity measure reflect situations where states had their generosity scores lowered by a half point because they had a spending cap but did not adjust for inflation.

We tested several alternative generosity measures including those that accounted for lifetime dollar caps, the presence or absence of ABA, no inflation adjustment, and more fine-grained age breakdowns. All produce similar patterns of results so we elected to go with the simplest specification. To demonstrate this robustness, an alternative (more complicated) generosity dependent variable specification is provided in Table H in S1 File. There we also present alternative modeling strategies analyzing the generosity of age caps alone, expenditure caps alone, and models with alternative independent variables included.

### Explanatory variables

To explain variations in ASD insurance mandate passage and generosity across states, we rely on several explanatory variables. The first two independent variables included in our analysis are designed to account for the role of politics in ASD benefit passage and generosity. As noted by Pitney (2015), ASD is inherently political, with high yearly treatment costs and an information environment that is “often murky, incomplete, interpretative, and open to manipulation” by the public and politicians [18]. The first measure in our analysis accounts for the partisanship of the state government in each state. Two points were awarded for Democratic control of the governor’s mansion and one point each were awarded for Democratic control of each chamber of the state legislature [80]. Thus, states with full Democratic control of state government are scored as 4 while states with unified Republican control are scored as zeros. Next, we include a measure of citizen ideology to account for the political preferences of the citizens. This yearly measure was originally developed by Berry et al. (1998), provides ideology data for all years from 2000–2016, and higher scores on the measure indicate a more liberal state populous [81]. While other measures of citizen ideology including those by Enns and Koch, Pacheco, and Caughey and Warshaw exist, we focus on the Berry ideology measure because it is the only one that provides relevant data throughout the twenty-first century [82–84].

In addition to accounting for the political environment in the state, it is also important to account for the relative policy need in each state for an ASD mandate. In other words, it is important to account for the number of individuals with ASD in each state. As a longitudinal count of the number of individuals with ASD across states is not publicly available, we use a proxy from the Department of Education providing a count of the number of children with ASD between the ages of 6 and 21 in each state-year receiving funds for special education through the IDEA Act. This child count is divided by the number of individuals in each state under the age of 20 to create our measure of policy need. Dividing by population size is necessary to account for differences in population size that might lead to higher ASD counts in large states. Models run using the ASD counts without accounting for population reveal an identical pattern of results. Critically, this autism child count data is only available from the US Department of Education beginning in 2005, and thus its influence cannot be assessed on the earliest adopting states.

Our models also include three measures of the state insurance environment. The first measure accounts for the percentage of health plans that are self-insured in each state-year, which comes from the Medical Expenditure Panel Survey. This measure is important to include because ERISA exempts self-insured plans from state insurance benefit legislation. In addition, our analysis includes measures for the percentage of state residents with employer-sponsored insurance and the percentage of state residents who are uninsured, both of which are available from U.S. Census Current Population Survey for our period of analysis. These measures are important to include because they serve as proxies for the insurance environment in the state. Notably, the percent uninsured measure helps to proxy for the prior generosity of the state towards its citizens in providing insurance coverage for those in need. States with a history of covering those in need of health coverage may be more predisposed to not only enact mandates for autism insurance coverage but also to ensure that they are generous.

The seventh independent variable in our analysis accounts for the economic circumstances within each state and is measured using median income. This measure is obtained from the Census bureau and is adjusted to 2016 dollars using the Consumer Price Index. Beyond economic circumstances, our study also includes two measures to account for the interest group environment in the state. Our variables come from Lowery et al. (2015) and are made public through the Michigan State Correlates of State Policy dataset. They separately measure the density of health interest groups and insurance interest groups in each state [85]. Specifically, the health interest group measure accounts for the number of health care interest groups registered in the state (i.e. health professional associations, hospitals, and health systems) and the insurance interest group measure accounts for the number of insurance interest groups in the state (i.e. representing insurance companies). These measures are only available for 1999 and 2007 so all state-years prior to 2007 are coded using 1999 data and all states starting in 2007 are coded using that data. While this lack of data across years is far from ideal, the Lowery et al. measures are regularly used in political science and health policy and better alternatives for health and insurance interest group influence are not available to our knowledge. We also attempted to include a specific measure in our analysis to account for the influence of Autism Speaks across the US states. Unfortunately, through direct communication with the organization, we learned that potential measures of influence including membership, donations, etc. are not available in the fifty state context.

Next, our study includes a measure of policy diffusion to account for the potential role of diffusion in the enactment and generosity of ASD insurance mandates. Our measure was developed in prior work by Chamberlain and Haider-Markel (2005) and Sylvester and Haider-Markel (2016). The measure of policy diffusion ranges from zero to one and accounts for the proportion of contiguous neighboring states that have adopted an autism insurance mandate as of a given state-year. Because Alaska and Hawaii have no actual neighbors, we considered Washington and Oregon as neighbors for Alaska. For Hawaii, we considered Oregon and California as neighboring states.

Lastly, the analysis includes a measure designed to account for the professionalism of the legislature in each state. Our legislative professionalism measure is a composite measure developed by Squire (2007) that accounts for key characteristics of the legislature including legislator salary, legislator resources, and session length [86]. While the Squire measure is the gold-standard for studying legislative professionalism, it is only updated roughly once every 5 years. For that reason, we also test a yearly legislative professionalism measure from Bowen and Greene (2014) in S1 File and find a similar pattern of results [67].

Data was collected for this project at the state-year level and includes observations for all 50 states from 2000–2017. Table A in S1 File presents summary information comparing the start and end date for each variable included in this manuscript, as well as details for how missing data for independent variables was managed.

### Method of analysis

Our analysis of ASD insurance mandates in the American states relies on either logistic regression or ordinal logistic regression (depending on dependent variable specification) including cubic polynomials for time and results clustered by state. In our initial models, each state enters our dataset in 2000 –before the passage of any mandates–and remains through their mandate’s passage sometime between 2001–2017. States that had not passed a mandate by the end of 2017 remain throughout our dataset. Thus, our dataset includes all state-years between 2000–2017 prior to mandate passage. Our modeling strategy relies on clustering to account for repeated state observations and we include cubic polynomials for time to account for temporal dependence within our observations [87]. Critically, our generosity results remain largely consistent across multiple alternative modeling strategies including using year fixed effects instead of cubic polynomials for time.

Our analysis proceeds as follows: we begin with descriptive statistics analyzing the spread of ASD mandates across the country and the generosity of benefits across states in Table 1. Then we move on to a multivariate analysis in Table 2 that uses logistic regression models to assess the influence of partisanship, economic circumstances, policy diffusion, the insurance environment, policy need, and legislative professionalism on the decision to enact an ASD insurance mandate. Then, in Tables 3 and 4 we use ordinal logistic regression to study the influence of these same explanatory variables on ASD mandate generosity. Specifically, Table 3 studies the predictors of the generosity of a state’s first autism insurance mandate and Table 4 presents results that keep states in our dataset until they have stopped modifying their autism insurance mandates. We analyze the generosity of first enactment (Table 3) separately from the generosity of all ASD mandates (Table 4) because the generosity of the initial mandate could be driven by a fundamentally different set of predictors then insurance mandate revision. Notably, all regression tables present separate models with and without the policy need measure included. This choice was made because the measure is only available from the Department of Education from 2005–2016, creating a large number of missing values in specifications including the measure. Tables in our paper present odds ratios but equivalent tables relying on log odds are available in the supplemental materials. Our data is available for download from the Texas Data Repository made available through Texas A&M University.

**Table 1:** The generosity of autism scores across states.

Autism Generosity Score	Number of States
0	4
0.5	9
1	5
1.5	9
2	12
2.5	3
3	5
4	3

**Table 2:** State predictors of passing an autism insurance mandate.

VARIABLES	Model 1	Model 2
	Any Mandate Provision	Any Mandate Provision
Dem. Govt. Control	1.40**	1.31*
	(0.213)	(0.205)
Citizen Ideology	1.04***	1.04***
	(0.015)	(0.016)
Median Household Income	0.99*	0.999
	(0.00004)	(0.00004)
Percent Uninsured	1.23**	1.19
	(0.126)	(0.127)
Percent Employer Sponsored	1.18**	1.14**
	(0.081)	(0.076)
Policy Diffusion	0.664	0.532
	(0.660)	(0.518)
Policy Need–# with ASD		2.86e-20
		(2.65e-18)
Percent Self Insured	1.02	1.01
	(0.040)	(0.040)
Interest Groups—Health	1.01**	1.01
	(0.006)	(0.006)
Interest Groups—Insurance	0.988	0.995
	(0.016)	(0.016)
Legislative Professionalism	.040*	0.077
	(0.071)	(.137)
T	0.478	68.27
	(0.263)	(255.95)
T2	1.14*	0.752
	(0.075)	(0.238)
T3	0.997	1.01
	(0.002)	(0.009)
Constant	2.25e-08	2.84e-15
	(9.45e-08)	(4.24e-14)
Observations	583	335
Pseudo R-Squared	0.30	0.23
Log Pseudolikelihood	-110.40	-99.29

Robust standard errors in parentheses

*** p<0.01,

** p<0.05,

* p<0.10

Results obtained using logistic regression including cubic polynomials for time and results clustered by state. The dependent variable is coded as 1 if a state mandate was passed in a given state-year and coded as zero otherwise. Where no mandate was enacted, data is updated to 2017. Presented results are odds ratios, models using log odds are available in S1 File.

**Table 3:** State predictors of the generosity of autism insurance mandates.

VARIABLES	Model 3: No Policy Need	Model 4: With Policy Need
	DV: Mandate Generosity	DV: Mandate Generosity
Dem. Govt. Control	1.40**	1.32*
	(0.220)	(0.220)
Citizen Ideology	1.04***	1.04**
	(0.015)	(0.016)
Median Household Income	0.99	0.999
	(0.00004)	(0.00004)
Percent Uninsured	1.21**	1.16
	(0.118)	(0.124)
Percent Employer Sponsored	1.17**	1.13*
	(0.076)	(0.073)
Policy Diffusion	0.531	0.437
	(0.532)	(0.436)
Policy Need–# with ASD		1.09e-16
		(9.26e-15)
Percent Self Insured	1.03	1.03
	(0.039)	(0.040)
Interest Groups—Health	1.01**	1.01
	(0.006)	(0.007)
Interest Groups—Insurance	0.988	0.994
	(0.015)	(0.016)
Legislative Professionalism	0.151	0.276
	(0.282)	(0.527)
T	0.443	54.61
	(0.233)	(202.69)
T2	1.15**	0.773
	(0.073)	(0.243)
T3	0.996	1.01
	(0.002)	(0.009)
Observations	583	335
Pseudo R-Squared	0.20	0.15
Log Pseudolikelihood	-192.14	-174.72

Robust standard errors in parentheses

*** p<0.01,

** p<0.05,

* p<0.10

Results obtained using ordinal logistic regression including cubic polynomials for time and results clustered by state. Where no mandate was enacted, data is updated to 2017. Presented results are odds ratios, models using log odds are available in S1 File. Results are robust to the removal of state clustering and several alternative modeling strategies which can be found in S1 File.

**Table 4:** Replication of Table 3 including all enactments, not just first enactment in states.

VARIABLES	Model 5: No Policy Need	Model 6: With Policy Need
	DV: Mandate Generosity	DV: Mandate Generosity
Dem. Govt. Control	1.30*	1.27*
	(0.178)	(0.184)
Citizen Ideology	1.04***	1.04***
	(0.013)	(0.015)
Median Household Income	1.00	0.99
	(0.00002)	(0.00003)
Percent Uninsured	1.06	1.09
	(0.075)	(0.091)
Percent Employer Sponsored	1.02	1.06
	(0.042)	(0.059)
Policy Diffusion	0.86	0.65
	(0.762)	(0.597)
Policy Need—# with ASD		8.16e+18
		(8.25e+20)
Percent Self Insured	1.04	1.03
	(0.035)	(0.036)
Interest Groups—Health	1.01*	1.01
	(0.007)	(0.008)
Interest Groups—Insurance	0.99	1.003
	(0.016)	(0.021)
Legislative Professionalism	0.03*	0.05
	(0.059)	(0.098)
T	0.36*	327.78
	(0.215)	(1453.23)
T2	1.20***	0.69
	(0.085)	(0.244)
T3	0.99**	1.01
	(0.003)	(0.009)
Observations	619	370
Pseudo R-Squared	0.25	0.19
Log Pseudolikelihood	-282.64	-256.03

Robust standard errors in parentheses

*** p<0.01,

** p<0.05,

* p<0.10

Results obtained using ordinal logistic regression including cubic polynomials for time and results clustered by state. In Table 3, states remain in the dataset until they pass their first autism insurance mandate and then they are removed on the assumption that first enactment is a different process than subsequent mandate revision. This table presents an alternative set of results keeping states in the dataset if they have subsequent mandate revisions beyond initial enactment until after all mandate revisions have occurred. Presented results are odds ratios, models using log odds are available in S1 File.

## Results

**Fig 1:**
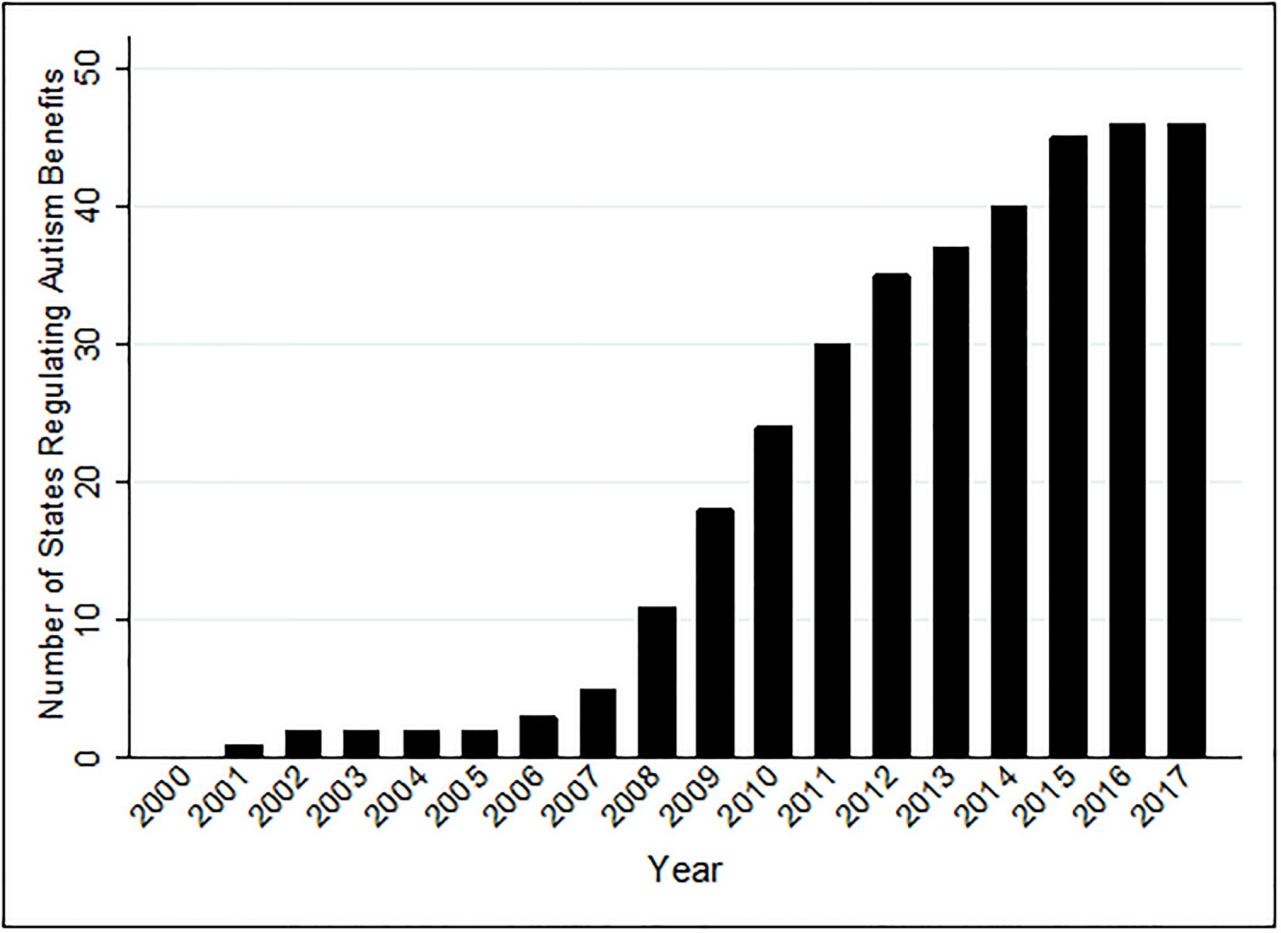
The enactment of autism insurance mandates over time.

When looking at the enactment of ASD insurance mandates across the country, several interesting patterns emerge. First, when looking at the growth of mandates across the US over time, it becomes clear that their passage is a distinctly twenty-first century phenomenon. The first ASD insurance mandate was signed into law in 2001 in Indiana and as seen in Fig 1, from 2001–2007, five states enacted mandates. In the past ten years however, the number of state mandates has expanded rapidly. Fig 1 shows that an additional thirteen states signed on in the next two years and that by the end of 2014, forty states had ASD mandates. By the end of 2017, forty-six states had signed mandates into law and Idaho, North Dakota, Washington, and Wyoming were the only holdouts. This pattern of results is rather remarkable. The adoption of any policy by 92% of the states in less than two decades is rare, and in the case of mandated benefit legislation, is unheard of [40].

It is also important to ask whether states are enacting generous mandated benefits or if the provisions being enacted in the states are relatively meager. We begin to explore this question in Table 1. There we find that states vary considerably in their placement in our generosity coding scheme but that states generally tilt towards less generous benefits. Eighteen states either have no insurance mandate or have a mandate that is not generous in terms of age restrictions or spending caps. Furthermore, another 42% of the states have scores of 1.5 or 2, indicating that they have a generous age limit or a generous spending cap, but not both. Only 11 states or 22% of the data falls into the two most generous categories of our dataset, indicating that most states place limitations on when individuals with ASD are eligible for insurance coverage that provides required treatment.

**Fig 2:**
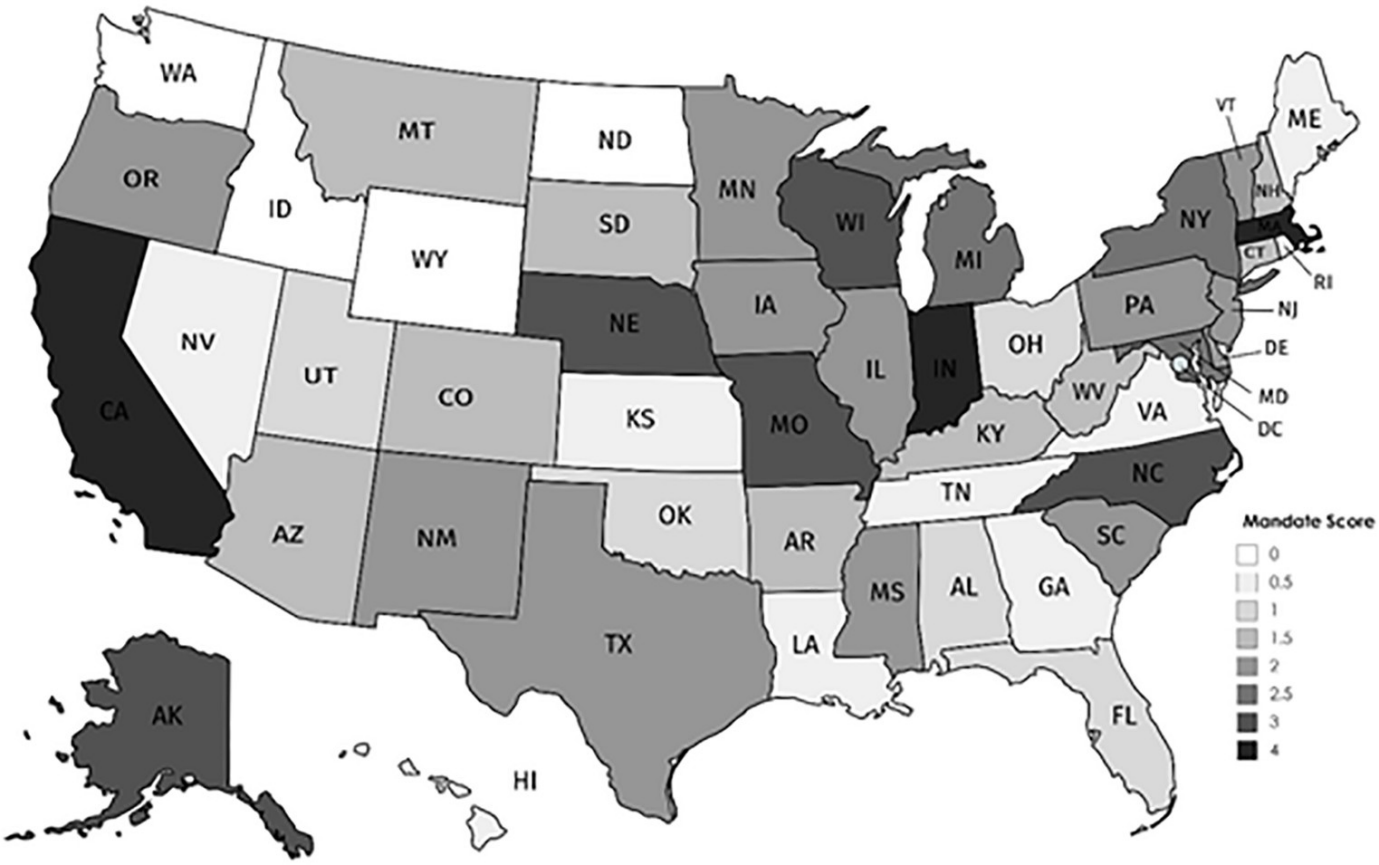
Geographic variation in the generosity of initial ASD insurance mandates, 2000–2017.

Table 1 helps us to understand the number of states that fall into each of our generosity categories but it provides little information about which states fall into each category. For that reason, our analysis also includes Fig 2, which presents a map of the fifty states with darker shading representing more generous ASD mandates. Fig 2 shows some interesting geographic patterns. First, we can see that the four states without ASD mandates are located in the Northwest, potentially indicating that the policy has not yet diffused to that region. Fig 2 also seems to provide mixed evidence for the potential role of partisanship in state generosity. On the one hand, it appears that many southern Republican states have meager benefits. On the other, states with the highest generosity scores seem to lack that clear partisan patterning. While the Democratic bastions of California and Massachusetts are scored as 4’s, so is less liberal Indiana. Ultimately, to sort out the true predictors of the enactment and generosity of ASD insurance mandates, a multivariate analysis is needed.

That multivariate analysis begins in Table 2 and reveals that partisanship and employer sponsored insurance are key predictors of states passing any ASD insurance mandate. Specifically, Model 1 in Table 2 suggests that increased Democratic control of state government makes states 1.40 times more likely to pass an ASD insurance mandate. Similarly, as citizen ideology becomes more liberal, states are 4% more likely to pass an ASD insurance mandate. Finally, a 1 percent increase in the number of individuals covered by employer sponsored health insurance makes states 1.18 times more likely to pass an ASD mandate.

Several other explanatory variables including median household income, percent uninsured, health interest groups, and legislative professionalism all also attain at least marginal significance in Model 1 of Table 2. That said, none of these findings are robust to the inclusion of our measure of policy need in Model 2 and thus should be interpreted with caution. Notably, our measures for policy diffusion, percent self-insured, and insurance interest groups are not significant in either model despite their theoretical relevance.

While understanding the predictors of passing an ASD insurance mandate is important, it is also critical to understand the drivers of the generosity of the ASD insurance mandates passed in each state. We begin to explore the drivers of this generosity in Table 3, which reveals a consistent pattern of results–that partisan dynamics drive variations in ASD mandate generosity while other theoretically relevant measures are less consistently influential. Specifically, in Model 3 –which presents the results of our generosity analysis without the autism policy need measure included–we can see that both Democratic control of state government and citizen ideology are positive and significant predictors of the generosity of initial ASD mandate generosity. Model 3 finds once again that a 1-unit increase in citizen ideology towards liberalism is associated with a 4% increase in the probability of passing a more generous mandate. Similarly, states with increased Democratic control of state government are more likely to enact generous mandates. In addition, Model 3 shows positive and significant relationships between percent uninsured, employer sponsored insurance, health interest groups, and generosity. This would suggest that a 1 percent increase in the uninsured rate, employer sponsored insurance, or a 1 unit increase in health interest groups increase the odds of a higher generosity score on enacted ASD mandates by 1.21, 1.17, and 1.01 times respectively.

While these findings are informative, it is important to acknowledge that Model 3 excludes a potentially critical explanatory variable due to its high degree of missing data in our early years of analysis–autism policy need. While the measure’s inclusion in Model 4 decreases the sample size by 248 observations, it reaffirms the importance of partisanship while calling other findings into question. In Model 4, Democratic control of state government and liberal citizen ideology remain positive and significant predictors of generous autism insurance mandates while the percent uninsured and health interest groups lose their significance in this model specification. Only percent employer sponsored insurance remains significant of the other significant factors from Model 1, suggesting that a 1 percent increase in employer sponsored insurance makes states 1.13 times more likely to enact a more generous mandate.

The analysis to this point has identified the importance of partisan measures to the passage of ASD mandates and the generosity of the initial ASD insurance mandate passed by states. That said, it is important to know if adding ASD mandate revisions by the legislature to the generosity dependent variable, which could make ASD mandates more or less generous over time, alters the dynamics presented in Table 3. For that reason, we also include Table 4, which replicates Table 3 while keeping states in our dataset until they have finished revising their ASD mandates. Our findings in Table 4 once again emphasize the importance of partisanship to state generosity. In Models 5 and 6 of Table 4, we find that Democratic control of state government and a liberal citizen population both increase the likelihood that ASD mandates will be more generous. Additionally, as was seen in Table 3, Table 4 finds a positive and marginally significant relationship between the presence of health interest groups and mandate generosity without, but not with, policy need included. Importantly however, once revisions to insurance mandates are included in the dependent variable, some key differences emerge. Most prominently, percent uninsured and percent with employer sponsored insurance are no longer significant predictors of mandate generosity. In addition, legislative professionalism achieves statistical significance for the first time. Specifically, we find that more professional state legislatures are less likely to enact generous ASD insurance mandates. That said, this finding should be interpreted with caution given its marginal significance and lack of significance in any model specification outside of Model 3.

## Discussion

Our analysis of ASD insurance mandates reveals several key findings for the study of mandated benefit legislation and ASD policy. First, our findings emphasize the importance of partisan factors and employer sponsored insurance to the enactment of ASD insurance mandates, regardless of generosity. Over the past two decades it appears that states have stepped forward to enact these mandates when they are under Democratic control of state government and as liberal citizen ideology increases. While this finding contradicts Johnson et al. (2014) who find that Republican states are more likely to enact ASD mandates, there several potential explanations for this difference. First, we believe that our findings differ because Johnson et al. (2014) stopped data collection in 2012, when only 30 states had passed ASD mandates. With that analysis capturing early adopting states like Indiana, South Carolina, and Texas but missing later mandate enactments by states like Hawaii, Delaware, Maryland, Minnesota, and Oregon, it is likely that the sign on party control of government has in fact flipped over time. The second reason could have been because of model specification strategies. While many of our measures overlap, our choice to include measures related to citizen ideology, legislative professionalism, policy diffusion, and the insurance environment could alter the finding identified in previous work.

Second, our paper is important to the study of ASD policy because our newly developed index of ASD mandate generosity demonstrates that there are substantial variations across states in the generosity of ASD benefits. Even as forty-six states took action from 2001–2017 to enact an insurance mandate, the scope of these benefits varies dramatically. A select number of states have mandated generous benefits, but we show that this is far from the norm. Instead, most states limit benefits by either restricting eligibility to individuals under a certain age or by capping the amount that insurance companies must spend to pay for needed behavioral treatments. Thus, our findings suggest that even as states have moved quickly to reform their policies, the number of individuals who are eligible for generous benefits varies dramatically.

More broadly, our identification and analysis of the differences in generosity of mandated benefit legislation represents an important new direction for future research on insurance mandates. To our knowledge, differences in the generosity of mandated benefit legislation has not been explored in depth in prior work. Given the wide variation seen on ASD policy however, and with prior research noting that similar variation exists on other topics [see 40, 50], future studies should explore this variation. In particular, scholars should analyze the predictors of mandated benefit legislation generosity on other public policies to test the durability of our findings here as well as to explore how differences in mandate generosity impact service utilization.

Beyond introducing the importance of generosity to the study of mandated benefit legislation, our analysis also highlights the key role of politics in the enactment of ASD policies in America. While the role of politics in the development of other parts of American health policy is well established, its role in the area of ASD policy is not yet well understood. Our analysis provides consistent evidence that not only does politics matter in this issue area, but also that it is the dominant predictor of benefit generosity for this vulnerable group. We show that increased Democratic control of state government makes states more likely to enact generous ASD mandates and that this finding holds while controlling for other common predictors of policy generosity. Just as important, we show that the political beliefs of state residents matter as well–controlling for the impact of the partisanship of politicians and other key factors, states with more liberal residents see more generous mandates enacted.

Beyond partisanship, percent of state residents with employer sponsored insurance and the number of health interest groups in states are also worth note. In models focusing on only first enactment (Table 3), states with more employees covered by private insurance increased the likelihood of adopting a generous mandate, potentially signaling that states move towards more generous benefits when there are more individuals the mandate will help. That said, as this measure only achieves standard levels (p <.05) of statistical significance in Model 1, and fails to approach statistical significance once mandate revisions are included in Table 4, this finding should be interpreted with caution. Furthermore, the positive and significant findings for health interest groups in Models 1 and 3 suggests that controlling for other factors including insurance interest groups, as the number of health interest groups goes up, so does ASD mandate generosity. That said, as with percent employer sponsored insurance, this interest group measure should be interpreted with caution as it loses statistical significance once policy need is added to the model.

Lastly, while there were strong theoretical reasons for the inclusion of our other explanatory measures, they rarely, if ever, were found to significantly impact ASD mandate generosity. Specifically, policy need, policy diffusion, economic circumstances, and insurance interest groups fail to ever achieve statistical significance and percent uninsured and legislative professionalism are only significant predictors of mandate generosity in one of four models. Taken together, our findings present a consistent picture that it is politics, not economics or potential need for an ASD mandate that drives mandate generosity.

This study makes several important contributions to our understanding of ASD policy in the US states. Nevertheless, the study has limitations that should be acknowledged. First, given the fast-pace at which state policies in this area have changed, our results cannot be considered final. States continue to innovate in the area of autism insurance mandates and future innovations could be driven by a different set of predictors then those identified as significant here.

Beyond a changing landscape, a second limitation with this paper is that while our analysis provides important information about the generosity of enacted state mandates, it does not account for differences in the implementation process across states. Mandated benefit legislation is quite complex and how bureaucratic officials, insurance companies, and other relevant parties interpret those statutes during implementation could alter their effects beyond the differences in generosity noted here. In addition, it remains possible that differences in the administrative capacity and bureaucratic oversight of insurance companies across states could alter the usage of ASD benefits, regardless of generosity levels. Future research should explore the implementation of mandated benefit legislation and its impact on the utilization of services across states.

Despite these limitations, we believe that our research represents an important step forward in our understanding of ASD policy in America. We have pointed to the fast-paced enactment of these mandates across states, the critical roles of political partisanship and ideology in this process, and the importance of accounting for generosity in the study of insurance mandates. We hope that this research will inspire future research on the connections between ASD policy and politics and between state legislative decisions on benefit generosity and service utilization.

## Supporting information

S1 File Online appendix final. | Table A. Variable Description Table. Table B. Replication of Table 2 Using Log Odds. Table C. Replication of Table 3 Using Log Odds. Table D. Replication of Table 4 Using Log Odds. Table E. Replication of Table 3 Using Yearly Bowen and Greene Leg. Professionalism Measure. Table F. Replication of Table 3 in Paper Without Clustering on State. Table G. Replication of Table 3 with Year Fixed Effects Instead of Polynomial Time Trends. Table H. Replication of Table 3 with Alternative DV Specification Discussed in S1 File. Table I. Replication of Table 4 with Alternative DV Specification Discussed in S1 File. Table J. Replication of Table 3 Interest Group Industry Measures with Generalized Interest Group Density. Table K. Replication of Table 3 –Model 2 Interacting Interest Group and Policy Need Measures. Table L. Continuous Alternative DVs–No Policy Need. Table M. Continuous Alternative DVs–With Policy Need.
(DOCX)

## References

[1] American Psychiatric Association. Diagnostic and statistical manual of mental disorders. 5th ed Arlington, VA: APA; 2013.

[2] Choi J, Lee S, Won J, Jin Y, Hong Y, Hur T-Y, et al Pathophysiological and neurobehavioral characteristics of a propionic acid-mediated autism-like rat model. PloS One 2018; 13(2): e0192925 10.1371/journal.pone.0192925 29447237 PMC5814017

[3] Centers for Disease Control and Prevention [Internet]. Austism spectrum disorder (ASD)—Data & statistics. https://www.cdc.gov/ncbddd/autism/data.html

[4] Chiri G, Warfield ME. Unmet need and problems accessing core health care services for children with autism spectrum disorder. Matern Child Health J 2012; 16(5): 1081–92. 10.1007/s10995-011-0833-6 21667201

[5] Buescher AV, Cidav Z, Knapp M, Mandell DS. Costs of autism spectrum disorder in the United Kingdom and the United States. JAMA Pediatrics 2014; 168(8): 721–28. 10.1001/jamapediatrics.2014.210 24911948

[6] Jacob A, Scott M, Falkmer M, Falkmer T. The costs and benefits of employing an adult with autism spectrum disorder: A systematic review. PloS One 2015; 10(1): e0139896. 26445345 10.1371/journal.pone.0139896 PMC4596848

[7] Mandell DS, Cao J, Ittenbach R, Pinto-Martin J. Medicaid expenditures for children with autistic spectrum disorders: 1994 to 1999. J Autism Dev Disord 2006; 36(4): 475–85. 10.1007/s10803-006-0088-z 16586155

[8] Ganz ML. The lifetime distribution of the incremental societal costs of autism. Arch Pediatr Adolesc Med. 2007; 161(4): 343–49. 10.1001/archpedi.161.4.343 17404130

[9] Johnson RA, Danis M, Hafner-Eaton C. US state variation in autism insurance mandates: Balancing access and fairness. Autism. 2014; 18(7): 803–14. 10.1177/1362361314529191 24789870 PMC4849527

[10] Leslie DL, Martin A. Health care expenditures associated with autism spectrum disorders. Arch Pediatr Adolesc Med. 2007; 161(4): 350–55. 10.1001/archpedi.161.4.350 17404131

[11] Silverman C. Understanding autism: Parents, doctors, and the history of a disorder. Princeton, NJ: Princeton University Press; 2011.

[12] Mandell DS, Barry CL, Marcus SC, Xie M, Shea K, Mullan K, et al Effects of autism spectrum disorder insurance mandates on the treated prevalence of autism spectrum disorder. JAMA Pediatrics 2016; 170(9): 887–93. 10.1001/jamapediatrics.2016.1049 27399053

[13] Autism Speaks [Internet]. State initiatives. https://www.autismspeaks.org/state-initiatives.

[14] Stuart EA, McGinty EE, Kalb L, Huskamp HA, Busch S, Gibson TB, et al Increased service use among children with autism spectrum disorder associated with mental health parity law. Health Affairs 2017; 36(2): 337–45. 10.1377/hlthaff.2016.0824 28167724 PMC8320748

[15] Harris SL, Handlemann JS. Age and IQ at intake as predictors of placement for young children with autism: A four-to six-year follow-up. J Autism Dev Disord 2000; 30(2): 137–42. 10832778 10.1023/a:1005459606120

[16] Kennedy-Hendricks A, Epstein AJ, Mandell DS, Candon MK, Marcus SC, Xie M, et al Effects of state autism mandate age caps on health service use and spending among adolescents. J Am Acad Child Adolesc Psychiatry 2018; 57(2): 125–31. 10.1016/j.jaac.2017.10.019 29413145 PMC5806145

[17] Baller JB, Barry CL, Shea K, Walker MM, Ouellette R, Mandell DS. Assessing early implementation of state autism insurance mandates. Autism 2016; 20(7): 796–807. 10.1177/1362361315605972 26614401 PMC4884164

[18] Pitney JJ Jr. The politics of autism: Navigating the contested spectrum. Lanham, MD: Rowman & Littlefield; 2015.

[19] Grace AM, Noonan KG, Cheng TL, Miller D, Verga B, Rubin D, et al The ACA’s pediatric essential health benefit has resulted in a state-by-state patchwork of coverage with exclusions. Health Affairs 2014; 33(12): 2136–43. 10.1377/hlthaff.2014.0743 25489031 PMC4587658

[20] Lavelle TA, Weinstein MC, Newhouse JP, Munir K, Kuhlthau KA, Prosser LA. Economic burden of childhood autism spectrum disorders. Pediatrics 2014; 133(3): 520–29. 10.1542/peds.2013-0763 PMC7034397 24515505

[21] Leigh JP, Grosse SD, Cassady D, Melnikow J, Hertz-Picciotto I. Spending by California’s department of developmental services for persons with autism across demographic and expenditure categories. PloS One 2016; 11(3): e0151970 10.1371/journal.pone.0151970 27015098 PMC4807877

[22] Stahmer AC, Mandell DS. State infant/toddler program policies for eligibility and services provision for young children with autism. Adm Policy Ment Health 2007; 34(1): 29–37. 10.1007/s10488-006-0060-4 16758329 PMC1764439

[23] Thompson C, Bolte S, Falkmer T, Girdler S. To be understood: Transitioning to adult life for people with autism spectrum disorder. PloS One 2018; 13(3): e0194758 10.1371/journal.pone.0194758 29579089 PMC5868819

[24] Marmor TR. The politics of autism: Navigating the contested spectrum. J Health Polit Policy Law 2017; 42(6): 1143–45.

[25] Stein BD, Sorbero MJ, Goswami U, Schuster J, Leslie DL. Impact of a private health insurance mandate on public sector autism service in Pennsylvania J Am Acad Child Adolesc Psychiatry 2012; 51(8): 771–79. 10.1016/j.jaac.2012.06.006 22840548

[26] Wang L, Mandell DS, Lawer L, Cidav Z, Leslie DL, Healthcare service use and costs for autism spectrum disorder: A comparison between Medicaid and private insurance. J Autism Dev Disord 2013; 43(5): 1057–64. 10.1007/s10803-012-1649-y 22965299 PMC3534815

[27] MacFarlane JR, Kanaya T. What does it mean to be autistic? Inter-state variation in special education criteria for autism services. J Child Fam Stud 2009; 18(6): 662–69.

[28] Turnbull HR, Wilcox BL, Stowe MJ. A brief overview of special education law with focus on autism. J Autism Dev Disord 2002; 32(5): 479–493. 12463521 10.1023/a:1020550107880

[29] Wei X, Wagner M, Christiano ER, Shattuck P, Yu JW. Special education services received by students with autism spectrum disorders from preschool through high school. J Spec Educ 2014; 48(3): 167–79. 10.1177/0022466913483576 25419002 PMC4235523

[30] Kohler FW. Examining the services received by young children with autism and their families: A survey of parent responses. Focus Autism Other Dev Disabl 1999; 14(3): 150–58.

[31] Kurth J, Mastergeorge AM. Individual education plan goals and services for adolescents with autism: Impact of age and educational setting. J Spec Educ 2010; 44(3): 146–60.

[32] White SW, Scahill L, Klin A, Koenig K, Volkmar FR. Educational placements and service use patterns of individuals with autism spectrum disorders. J Autism Dev Disord 2007; 37(8): 1403–12. 10.1007/s10803-006-0281-0 17082975

[33] Bouder JN, Spielman S, Mandell DS. Brief report: Quantifying the impact of autism coverage on private insurance premiums. J Autism Dev Disord 2009; 39(6): 963–57. 10.1007/s10803-009-0701-z PMC2862974 19214727

[34] Zane T, Davis C, Rosswurm M. The cost of fad treatments in autism. J Early Intensive Behav Interv 2008; 5(2): 44–51.

[35] Autism Speaks [Internet]. Autism law summit resource hub. https://www.autismlawsummit.com/resource-hub.

[36] Centers for Disease Control and Prevention. [Internet] Autism spectrum disorder (ASD)—Treatment. https://www.cdc.gov/ncbddd/autism/treatment.html.

[37] Frank RG, Koyanagi C, McGuire TG. The politics and economics of mental health parity laws. Health Affairs 1997; 16(4): 108–19. 9248154 10.1377/hlthaff.16.4.108

[38] Unumb L. Personal interview by phone with autism speaks official. June 29, 2017.

[39] Berry KN, Huskamp HA, Goldman HH, Rutkow L, Barry CL. Litigation provides clues to ongoing challenges in implementing insurance parity. J Health Polit Policy Law 2017; 42(6): 1065–98. 10.1215/03616878-4193630 28801470

[40] Laugesen MJ, Paul RR, Luft HS, Aubry W, Ganiats TG. A comparative analysis of mandated benefit laws, 1949–2002. Health Serv Res 2006; 41(2): 1081–103. 16704673 10.1111/j.1475-6773.2006.00521.x PMC1713218

[41] Department of Health and Human Services [Internet]. Health benefits & coverage: What marketplace health insurance plans cover. https://www.healthcare.gov/coverage/what-marketplace-plans-cover/.

[42] Nathenson R. Coverage mandates and market dynamics: Employer, insurer, and patient responses to parity laws. Health Econ Policy Law 2018; 10 (12): 10.1017/S1744133118000294 30309399

[43] Bagley N, Levy H. Essential health benefits and the Affordable Care Act: Law and process. J Health Polit Policy Law 2014; 39(2): 441–65. 10.1215/03616878-2416325 24305849 PMC4116669

[44] Cason M [Internet]. Bill to require autism insurance coverage changed. http://www.al.com/news/birmingham/index.ssf/2017/05/bill_to_require_autism-insuran.html.

[45] Petroski, W [Internet]. Iowa legislature backs insurance coverage for autism. https://www.desmoinesregister.com/story/news/politics/2017/03/23/iowa-legislature-backs-insurance-coverage-autism/99539772/.

[46] Campbell C [Internet]. New insurance mandate for autism treatment lifts families’ financial burden. http://www.newsobserver.com/news/politics-government/state-politics/article60119656.html.

[47] Sabik LM, Laugesen MJ. The impact of maternity length-of-stay mandates on the labor market and insurance coverage. Inquiry 2012; 49(1): 37–51. 10.5034/inquiryjrnl_49.01.05 22650016

[48] Jensen GA, Morrisey MA. Employer-sponsored health insurance and mandated benefit laws. Milbank Q 1999; 77(4): 425–59. 10656028 10.1111/1468-0009.00147 PMC2751135

[49] Gabel JR, Jensen GA. The price of state mandated benefits. Inquiry 1989; 26(4): 419–31. 2533169

[50] Schauffler HH. Politics trumps science: Rethinking state-mandated benefits. Am J Prev Med 2000; 19(2): 136–37. 10913906 10.1016/s0749-3797(00)00190-2

[51] Summers LH. Some simple economics of mandated benefits. Am Econ Rev 1989; 79(2): 177–83.

[52] Schmidt L. Effects of infertility insurance mandates on fertility. J Health Econ 2007; 26(3): 431–46. 10.1016/j.jhealeco.2006.10.012 17129624 PMC2096618

[53] Rathore SS, McGreevey JD, Schulman KA, Atkins D. Mandated coverage for cancer-screening services: Whose guidelines do states follow?. Am J Prev Med 2000; 19(2): 71–78. 10913895 10.1016/s0749-3797(00)00179-3

[54] Andersen M. Heterogeneity and the effect of mental health parity mandates on the labor market. J Health Econ 2015; 43(1): 74–84. 26210944 10.1016/j.jhealeco.2015.06.008 PMC4591173

[55] Klick J, Markowitz. Are mental health insurance mandates effective? Evidence from suicides. Health Econ 2006; 15(1): 83–97. 10.1002/hec.1023 16145720

[56] Bafumi J, Shapiro RY. A new partisan voter. J Polit 2009; 71(1): 1–24.

[57] Callaghan T, Jacob LR. The future of health care reform: What is driving enrollment? J Health Polit Policy Law 2016; 42(2): 215–46. 10.1215/03616878-3766710 28007795

[58] Oberlander J. Under-siege: The individual mandate for health insurance and its alternatives. N Engl J Med 2011; 364(March 11): 1085–87. 21323536 10.1056/NEJMp1101240

[59] Jacobs LR, Callaghan T. Why states expand Medicaid: Party, resources, and history. J Health Polit Policy Law 2013; 38(5): 1023–50. 10.1215/03616878-2334889 23794741

[60] Rigby E. Haselswerdt J. Hybrid federalism, partisan politics, and early implementation of state health insurance exchanges. Publius 2013; 43(3): 368–91.

[61] Frean M, Gruber J, Sommers BD. Premium subsidies, the mandate, and Medicaid expansion: Coverage effects of the Affordable Care Act. Cambridge, MA: National Bureau of Economic Research; 2016. 10.1016/j.jhealeco.2017.02.004 28319791

[62] Jacobs LR, Scocpol T. Health care reform and American politics: What everyone needs to know, 3^rd^ edition New York, NY: Oxford University Press; 2016.

[63] Howard C. The welfare state nobody knows: Debunking mythis about US social policy. Princeton, NJ: Priceton University Press; 2008.

[64] Welch S, Thompson K. The impact of federal incentives on state policy innovation. Am J Pol Sci 1980; 24(4): 715–29.

[65] Davies G, Derthick M. Race and social welfare policy: The Social Security Act of 1935. Polit Sci Q 1997; 113(2): 217–35.

[66] Boushey G. Policy diffusion dynamics in America. New York, NY: Cambridge University Press; 2010.

[67] Bowen DC, Greene Z. Should we measure professionalism with an index? A note on theory and practice in state leglislative professionalism research. State Polit Policy Q 2014; 14(3): 277–96.

[68] Lax JR, Phillips JH. The democratic deficit in the states. Am J Pol Sci 2012; 56(1): 148–66.

[69] Squire P. Professionalization and public opinion of state legislatures. J Polit 1993; 55(2): 479–91.

[70] Squire P. Legislative professionalization and membership diversity in state legislatures. Legislative Studies Quarterly 1992; 17(1): 69–79.

[71] Schlozman KL, Tierney JT. Organized interests and American democracy. New York, NY: Harper & Row; 1986.

[72] Gray V, Lowery D, Benz J. Organized interests and health policy in the American states. Washington, DC: Georgetown University Press; 2013.

[73] Boushey G. Policy diffusion dynamics in America. New York, NY: Cambridge University Press; 2010.

[74] Walker JL. The diffusion of innovations among the United States. Am Polit Sci Rev 1969; 63(3): 880–99.

[75] Berry FS, Berry WD. State lottery adoptions as policy innovations: An event history analysis. Am Polit Sci Rev 1990; 84(2): 395–415.

[76] Haider-Markel DP, Meier K. The politics of gay and lesbian rights: Expanding the scope of conflict. J Polit 1996; 58(2): 332–49.

[77] Sylvester SM, Haider-Markel DP. Buzz kill: State adoption of DUI interlock laws, 2005–11. Policy Stud J 2016; 44(4): 491–509.

[78] Volden C. States as policy laboratories: Emulating success in the children’s health insurance program. Am J Pol Sci 2006; 50(2): 294–312.

[79] National Conference of State Legislatures [Internet]. Autism and insurance coverage state laws. http://www.nscl.org/research/health/autism-and-insurance-coverage-state-laws.aspx.

[80] Rocco P, Kelly AS, Keller AC. Politics at the cutting edge: Intergovernmental policy innovation in the Affordable Care Act. Publius 2018; 48(3): 425–53.

[81] Berry WD, Ringquist EJ, Fording RC, Hanson RL. Measuring citizen and government ideology in the American states: 1960–1993. Am J Pol Sci 1998; 42(1): 327–48.

[82] Enns PK, Koch J. Public opinion in the US states: 1956 to 2010. State Polit Policy Q 2013; 13(3): 349–72.

[83] Caughey D, Warshaw C. The dynamics of state policy liberalism, 1936–2014. Am J Pol Sci 2016; 60(4): 899–913.

[84] Paceheco J. The thermostatic model of responsiveness in the American states. State Polit Policy Q 2013; 13(3): 306–32.

[85] Lowery D, Gray V, Cluverius J. Temporal change in the density of state interest communities: 1980 to 2007. State Polit Policy Q 2015; 15(2): 263–86.

[86] Squire P. Measuring state legislative professionalism: The squire index revisited. State Polit Policy Q 2007; 7(2): 211–27.

[87] Carter DB, Signorino CS. Back to the future: Modeling time dependence in binary data. Polit Anal 2010; 18(3): 271–92.

