# Supplementary material for: Autism spectrum disorder, politics, and the generosity of insurance mandates in the United States
Source: PLoS One. 2019 May 24;14(5):e0217064. doi: 10.1371/journal.pone.0217064 (PMC6534322; doi:10.1371/journal.pone.0217064)
Supplement: S1 File — | Table A. Variable Description Table. Table B. Replication of Table 2 Using Log Odds. Table C. Replication of Table 3 Using Log Odds. Table D. Replication of Table 4 Using Log Odds. Table E. Replication of Table 3 Using Yearly Bowen and Greene Leg. Professionalism Measure. Table F. Replication of Table 3 in Paper Without Clustering on State. Table G. Replication of Table 3 with Year Fixed Effects Instead of Polynomial Time Trends. Table H. Replication of Table 3 with Alternative DV Specification Discussed in S1 File. Table I. Replication of Table 4 with Alternative DV Specification Discussed in S1 File. Table J. Replication of Table 3 Interest Group Industry Measures with Generalized Interest Group Density. Table K. Replication of Table 3 –Model 2 Interacting Interest Group and Policy Need Measures. Table L. Continuous Alternative DVs–No Policy Need. Table M. Continuous Alternative DVs–With Policy Need. (DOCX) [file pone.0217064.s001.docx]

Appendix A: Dependent Variable Descriptions

Autism Insurance Mandate Generosity Dependent Variable

- 1 point – Any autism insurance mandate
- 1 point – mandate covers adults
- 1 point – mandate spending cap above $36,000 median spending cap
- -.5 points – no inflation adjustment if spending cap present
- 1 additional point – Broad coverage – no age or spending cap (Makes highest possible score 4)

Note: In states with hour caps instead of spending caps, hour caps were converted to spending caps using established method of Autism Speaks in legislative debates over blended rates which results in a rate of $30.87 dollars per hour. This assumes a combination of consultant and line therapist reimbursement rates. While some variation in cost is inherent across states, this is the only established rate widely used in debates. The formulation of this rate is available upon request.

Alternative Insurance mandate Generosity Dependent Variable – Used in Appendix

- 2 points – Any autism insurance mandate
- 1 point – mandate covers teenagers
- 1 point – mandate covers adults
- 1 point – ABA included in mandate
- 1 point - mandate spending cap above $36,000 median spending cap
- - 1 point – No inflation adjustment
- - 1 point – Lifetime cap on spending
- 1 additional point – Broad coverage – no age or spending cap (Makes highest possible score 7)

Note: Other alternative specifications were tested varying the point breakdowns noted here and the same pattern of results was found again and again.

***A Note on Tennessee and Washington State:***

It is critical to acknowledge that some disagreement exists over the mandate status of Tennessee and Washington State. We code Tennessee as having passed a weak mandate based on legislation identified by the National Conference of State Legislatures that requires insurers providing coverage for neurological disorders to provide coverage for ASD. Autism Speaks however, views this as not meeting the threshold for a mandate. Alternatively, we code Washington State as not having passed a mandate because no mandate has been passed into law in the state. That said, Autism Speaks considers Washington to have generous benefits that were achieved through litigation as opposed to legislation. Robustness tests that code Tennessee as a zero or that treat Washington State as a missing observation given the legislation vs. litigation issue from this study identify no changes in the pattern of results we present here.

Appendix B: Additional Tables and Alternative Modeling Strategies

Table A. Variable Description Table

| Variable | Years Available | Additional Notes |
| --- | --- | --- |
| Generosity | 2000-2017 | N/A |
| Citizen Ideology | 2000-2016 | 2016 data used for 2017 |
| Democratic Govt. Control | 2000-2017 | N/A |
| Median Household Income | 2000-2016 | 2016 data used for 2017 |
| Percent Uninsured | 2000-2017 | N/A |
| Percent Employer Sponsored Insurance | 2000-2017 | N/A |
| Policy Need – Children with ASD | 2005-2016 | No data prior to 2005 – treated as missing |
| Percent Self Insured | 2000-2006; 2008-2017 | Missing values filled in with most recent available year; including all of 2007 taken from 2006 |
| Interest Groups - Health | 1999; 2007 | 1999 data used 2000-2006; 2007 data used thereafter |
| Interest Groups - Insurance | 1999; 2007 | 1999 data used 2000-2006; 2007 data used thereafter |
| Squire Legislative Prof. | 1996; 2003; 2009; 2015 | Most recently available year used |
| Bowen and Greene Leg. Prof. | 2000-2014 (biennial) | Biennial data; 2013/2014 legislative session data used for 2015-2017 |
| Policy Diffusion | 2000-2017 | N/A |

Table B. Replication of Table 2 using Log Odds

|  | Model 1 | Model 2 |
| --- | --- | --- |
| VARIABLES | Any Mandate Provision | Any Mandate Provision |
|  |  |  |
| Dem. Govt. Control | 0.33** | 0.27* |
|  | (0.153) | (0.156) |
| Citizen Ideology | 0.04*** | 0.04*** |
|  | (0.015) | (0.016) |
| Median Household Income | -0.00007* | -0.00006 |
|  | (0.00004) | (0.00004) |
| Percent Uninsured | 0.21** | 0.17 |
|  | (0.102) | (0.107) |
| Percent Employer Sponsored | 0.17** | 0.13** |
|  | (0.069) | (0.067) |
| Policy Diffusion | -0.41 | -0.63 |
|  | (0.993) | (0.972) |
| Policy Need – # with ASD |  | -45.00 |
|  |  | (92.587) |
| Percent Self Insured | 0.02 | 0.01 |
|  | (0.039) | (0.039) |
| Interest Groups - Health | 0.01** | 0.01 |
|  | (0.006) | (0.006) |
| Interest Groups - Insurance | -0.01 | -0.01 |
|  | (0.016) | (0.016) |
| Legislative Professionalism | -3.21* | -2.57 |
|  | (1.753) | (1.785) |
| T | -0.74 | 4.22 |
|  | (0.551) | (3.749) |
| T2 | 0.13* | -0.29 |
|  | (0.066) | (0.317) |
| T3 | -0.003 | 0.01 |
|  | (0.002) | (0.009) |
| Constant | -17.61*** | -33.50** |
|  | (4.195) | (14.960) |
|  |  |  |
| Observations | 583 | 335 |
| Pseudo R-Squared | 0.30 | 0.23 |
| Log Pseudolikelihood | -110.40 | -99.29 |

Robust standard errors in parentheses

*** p<0.01, ** p<0.05, * p<0.10

NOTES: Results obtained using logistic regression including cubic polynomials for time and results clustered by state. The dependent variable is coded as 1 if a state mandate was passed in a given state-year and coded as zero otherwise. Where no mandate was enacted, data is updated to 2017. Presented results are log odds.

Table C. Replication of Table 3 using Log Odds

|  | Model 3: No Policy Need | Model 4: With Policy Need |
| --- | --- | --- |
| VARIABLES | DV: Mandate Generosity | DV: Mandate Generosity |
|  |  |  |
| Dem. Govt. Control | 0.34** | 0.28* |
|  | (0.157) | (0.166) |
| Citizen Ideology | 0.04*** | 0.04** |
|  | (0.014) | (0.016) |
| Median Household Income | -0.00006 | -0.00006 |
|  | (0.00004) | (0.00004) |
| Percent Uninsured | 0.19** | 0.15 |
|  | (0.097) | (0.107) |
| Percent Employer Sponsored | 0.16** | 0.12* |
|  | (0.065) | (0.064) |
| Policy Diffusion | -0.63 | -0.83 |
|  | (1.002) | (0.997) |
| Policy Need – # with ASD |  | -36.75 |
|  |  | (84.715) |
| Percent Self Insured | 0.03 | 0.03 |
|  | (0.038) | (0.038) |
| Interest Groups - Health | 0.01** | 0.01 |
|  | (0.006) | (0.007) |
| Interest Groups - Insurance | -0.01 | -0.01 |
|  | (0.015) | (0.016) |
| Legislative Professionalism | -1.89 | -1.29 |
|  | (1.862) | (1.911) |
| T | -0.81 | 4.00 |
|  | (0.525) | (3.712) |
| T2 | 0.14** | -0.26 |
|  | (0.063) | (0.314) |
| T3 | -0.004* | 0.01 |
|  | (0.002) | (0.009) |
|  |  |  |
| Observations | 583 | 335 |
| Pseudo R-Squared | 0.20 | 0.15 |
| Log Pseudolikelihood | -192.14 | -174.72 |

Robust standard errors in parentheses

*** p<0.01, ** p<0.05, * p<0.10

NOTES: Results obtained using ordinal logistic regression including cubic polynomials for time and results clustered by state. Where no mandate was enacted, data is updated to 2017. Presented results are log odds. Results are robust to the removal of state clustering and several alternative modeling strategies which can be found in the Appendix.

Table D. Replication of Table 4 Using Log Odds

|  | Model 5: No Policy Need | Model 6: With Policy Need |
| --- | --- | --- |
| VARIABLES | DV: Mandate Generosity | DV: Mandate Generosity |
|  |  |  |
| Dem. Govt. Control | 0.26* | 0.24* |
|  | (0.137) | (0.144) |
| Citizen Ideology | 0.04*** | 0.04*** |
|  | (0.012) | (0.014) |
| Median Household Income | 5.07e-06 | -0.00002 |
|  | (0.00002) | (0.00002) |
| Percent Uninsured | 0.06 | 0.08 |
|  | (0.071) | (0.084) |
| Percent Employer Sponsored | 0.02 | 0.06 |
|  | (0.041) | (0.056) |
| Policy Diffusion | -0.15 | -0.44 |
|  | (0.886) | (0.925) |
| Policy Need – # with ASD |  | 43.55 |
|  |  | (101.127) |
| Percent Self Insured | 0.04 | 0.03 |
|  | (0.034) | (0.035) |
| Interest Groups - Health | 0.01* | 0.01 |
|  | (0.007) | (0.008) |
| Interest Groups - Insurance | -0.00 | 0.00 |
|  | (0.016) | (0.021) |
| Legislative Professionalism | -3.48* | -3.05 |
|  | (1.918) | (2.063) |
| T | -1.01* | 5.79 |
|  | (0.590) | (4.434) |
| T2 | 0.18** | -0.38 |
|  | (0.071) | (0.355) |
| T3 | -0.01** | 0.01 |
|  | (0.003) | (0.009) |
|  |  |  |
| Observations | 619 | 370 |
| Pseudo R-Squared | 0.25 | 0.19 |
| Log Pseudolikelihood | -282.64 | -256.03 |

Robust standard errors in parentheses

*** p<0.01, ** p<0.05, * p<0.10

Note: Results obtained using ordinal logistic regression including cubic polynomials for time and results clustered by state. In Table 3, states remain in the dataset until they pass their first autism insurance mandate and then they are removed on the assumption that first enactment is a different process than subsequent mandate revision. This table presents an alternative set of results keeping states in the dataset if they have subsequent mandate revisions beyond initial enactment until after all mandate revisions have occurred. Presented results are log odds.

Table E. Replication of Table 3 using Yearly Bowen and Greene Leg. Professionalism Measure

|  | Model 3: No Policy Need | Model 4: With Policy Need |
| --- | --- | --- |
| VARIABLES | DV: Mandate Generosity | DV: Mandate Generosity |
|  |  |  |
| Dem. Govt. Control | 0.33** | 0.27 |
|  | (0.159) | (0.170) |
| Citizen Ideology | 0.04*** | 0.04*** |
|  | (0.015) | (0.016) |
| Median Household Income | -0.00005 | -0.00005 |
|  | (0.00004) | (0.00004) |
| Percent Uninsured | 0.21** | 0.17 |
|  | (0.096) | (0.106) |
| Percent Employer Sponsored | 0.15** | 0.12* |
|  | (0.065) | (0.065) |
| Policy Diffusion | -0.63 | -0.78 |
|  | (0.990) | (0.984) |
| Policy Need – # with ASD |  | -44.56 |
|  |  | (91.418) |
| Percent Self Insured | 0.03 | 0.02 |
|  | (0.038) | (0.038) |
| Interest Groups - Health | 0.01* | 0.01 |
|  | (0.006) | (0.006) |
| Interest Groups - Insurance | -0.01 | -0.00 |
|  | (0.014) | (0.016) |
| Legislative Professionalism | -0.14 | -0.09 |
|  | (0.157) | (0.148) |
| T | -0.89* | 3.59 |
|  | (0.530) | (3.593) |
| T2 | 0.15** | -0.22 |
|  | (0.064) | (0.306) |
| T3 | -0.00* | 0.01 |
|  | (0.002) | (0.008) |
|  |  |  |
| Observations | 566 | 332 |
| Pseudo R-Squared | 0.20 | 0.15 |
| Log Pseudolikelihood | -183.59 | -166.44 |

Robust standard errors in parentheses

*** p<0.01, ** p<0.05, * p<0.10

NOTES: Results obtained using ordinal logistic regression including cubic polynomials for time and results clustered by state. Where no mandate was enacted, data is updated to 2017. This Table replaces the Squire legislative professionalism measure from Table 3 of the paper with Bowen and Greene’s measure, which is updated for each legislative session.

Table F. Replication of Table 3 in Paper Without Clustering on State

|  | Model 3: No Policy Need | Model 4: With Policy Need |
| --- | --- | --- |
| VARIABLES | DV: Mandate Generosity | DV: Mandate Generosity |
|  |  |  |
| Dem. Govt. Control | 0.34** | 0.28* |
|  | (0.139) | (0.143) |
| Citizen Ideology | 0.04** | 0.04** |
|  | (0.017) | (0.017) |
| Median Household Income | -0.00006 | -0.00006 |
|  | (0.00004) | (0.00004) |
| Percent Uninsured | 0.19** | 0.15 |
|  | (0.094) | (0.099) |
| Percent Employer Sponsored | 0.16** | 0.12* |
|  | (0.065) | (0.067) |
| Policy Diffusion | -0.63 | -0.83 |
|  | (0.845) | (0.848) |
| Policy Need – # with ASD |  | -36.75 |
|  |  | (132.488) |
| Percent Self Insured | 0.03 | 0.03 |
|  | (0.044) | (0.045) |
| Interest Groups - Health | 0.01** | 0.01* |
|  | (0.005) | (0.006) |
| Interest Groups - Insurance | -0.01 | -0.01 |
|  | (0.014) | (0.015) |
| Legislative Professionalism | -1.89 | -1.29 |
|  | (2.129) | (2.184) |
| T | -0.81 | 4.00 |
|  | (0.655) | (3.903) |
| T2 | 0.14* | -0.26 |
|  | (0.078) | (0.333) |
| T3 | -0.004 | 0.01 |
|  | (0.003) | (0.009) |
|  |  |  |
| Observations | 583 | 335 |
| Pseudo R-Squared | 0.20 | 0.15 |
| Log Pseudolikelihood | -192.14 | -174.72 |

Standard errors in parentheses

*** p<0.01, ** p<0.05, * p<0.10

NOTES: Results obtained using ordinal logistic regression including cubic polynomials for time. Where no mandate was enacted, data is updated to 2017. This Table replicates from Table 3 of the paper while eliminating clustering by state.

Table G. Replication of Table 3 with Year Fixed Effects Instead of Polynomial Time Trends

|  | Model 3: No Policy Need | Model 4: With Policy Need |
| --- | --- | --- |
| VARIABLES | DV: Mandate Generosity | DV: Mandate Generosity |
|  |  |  |
| Dem. Govt. Control | 0.33** | 0.28* |
|  | (0.159) | (0.169) |
| Citizen Ideology | 0.04** | 0.04** |
|  | (0.016) | (0.017) |
| Median Household Income | -0.00006 | -0.00006 |
|  | (0.00004) | (0.00004) |
| Percent Uninsured | 0.17 | 0.15 |
|  | (0.114) | (0.121) |
| Percent Employer Sponsored | 0.16** | 0.13 |
|  | (0.078) | (0.079) |
| Policy Diffusion | -0.93 | -0.96 |
|  | (0.944) | (0.953) |
| Policy Need – # with ASD |  | -19.55 |
|  |  | (92.960) |
| Percent Self Insured | 0.04 | 0.03 |
|  | (0.039) | (0.040) |
| Interest Groups - Health | 0.01* | 0.01 |
|  | (0.007) | (0.007) |
| Interest Groups - Insurance | -0.01 | -0.01 |
|  | (0.016) | (0.017) |
| Legislative Professionalism | -2.12 | -1.65 |
|  | (2.129) | (2.263) |
|  |  |  |
| Observations | 583 | 335 |
| Pseudo R-Squared | 0.22 | 0.15 |
| Log Pseudolikelihood | -186.94 | -172.92 |

Robust standard errors in parentheses

*** p<0.01, ** p<0.05, * p<0.10

NOTES: Results obtained using ordinal logistic regression, results clustered by state, and year fixed effects instead of polynomial time trends. Year fixed effects are excluded from the regression output to allow the results to fit onto a single page. Where no mandate was enacted, data is updated to 2017. We have also modeled an alternative specification including year fixed effects and no state clustering. That model specification is available upon request.

Table H. Replication of Table 3 with Alternative DV Specification Discussed in Appendix A

|  | Model 3: No Policy Need | Model 4: With Policy Need |
| --- | --- | --- |
| VARIABLES | DV: Mandate Generosity | DV: Mandate Generosity |
|  |  |  |
| Dem. Govt. Control | 0.36** | 0.30* |
|  | (0.161) | (0.169) |
| Citizen Ideology | 0.04*** | 0.04** |
|  | (0.015) | (0.016) |
| Median Household Income | -0.00006 | -0.00006 |
|  | (0.00004) | (0.0004) |
| Percent Uninsured | 0.20** | 0.16 |
|  | (0.099) | (0.107) |
| Percent Employer Sponsored | 0.17** | 0.14** |
|  | (0.066) | (0.064) |
| Policy Diffusion | -0.70 | -0.89 |
|  | (1.020) | (1.009) |
| Policy Need – # with ASD |  | -32.12 |
|  |  | (86.693) |
| Percent Self Insured | 0.04 | 0.03 |
|  | (0.038) | (0.039) |
| Interest Groups - Health | 0.01** | 0.01 |
|  | (0.006) | (0.007) |
| Interest Groups - Insurance | -0.01 | -0.01 |
|  | (0.015) | (0.016) |
| Legislative Professionalism | -1.94 | -1.36 |
|  | (1.903) | (1.925) |
| T | -0.88 | 3.78 |
|  | (0.534) | (3.705) |
| T2 | 0.15** | -0.24 |
|  | (0.065) | (0.314) |
| T3 | -0.004* | 0.01 |
|  | (0.002) | (0.009) |
|  |  |  |
| Observations | 583 | 335 |
| Pseudo R-Squared | 0.21 | 0.15 |
| Log Pseudolikelihood | -185.03 | -170.41 |

Robust standard errors in parentheses

*** p<0.01, ** p<0.05, * p<0.10

NOTES: Results obtained using ordinal logistic regression including cubic polynomials for time and results clustered by state. Where no mandate was enacted, data is updated to 2017. This Table replicates Table 3 of the paper while using the alternative DV specification discussed in in the supporting information.

Table I. Replication of Table 4 with Alternative DV Specification Discussed in Appendix A

|  | Model 5: No Policy Need | Model 6: With Policy Need |
| --- | --- | --- |
| VARIABLES | DV: Mandate Generosity | DV: Mandate Generosity |
|  |  |  |
| Dem. Govt. Control | 0.33** | 0.31** |
|  | (0.142) | (0.148) |
| Citizen Ideology | 0.04*** | 0.04*** |
|  | (0.012) | (0.014) |
| Median Household Income | -4.58e-07 | -0.00002 |
|  | (0.00003) | (0.00003) |
| Percent Uninsured | 0.05 | 0.09 |
|  | (0.074) | (0.086) |
| Percent Employer Sponsored | 0.03 | 0.07 |
|  | (0.043) | (0.056) |
| Policy Diffusion | -0.43 | -0.73 |
|  | (0.917) | (0.938) |
| Policy Need – # with ASD |  | 57.28 |
|  |  | (107.462) |
| Percent Self Insured | 0.03 | 0.02 |
|  | (0.035) | (0.035) |
| Interest Groups - Health | 0.01 | 0.01 |
|  | (0.006) | (0.008) |
| Interest Groups - Insurance | 0.00 | 0.01 |
|  | (0.016) | (0.020) |
| Legislative Professionalism | -3.17* | -2.67 |
|  | (1.815) | (1.902) |
| T | -1.02* | 5.19 |
|  | (0.596) | (4.099) |
| T2 | 0.19*** | -0.32 |
|  | (0.072) | (0.329) |
| T3 | -0.01** | 0.01 |
|  | (0.003) | (0.009) |
|  |  |  |
| Observations | 619 | 370 |
| Pseudo R-Squared | 0.24 | 0.19 |
| Log Pseudolikelihood | -290.68 | -266.19 |

Robust standard errors in parentheses

*** p<0.01, ** p<0.05, * p<0.10

NOTES: Results obtained using ordinal logistic regression including cubic polynomials for time and results clustered by state. Where no mandate was enacted, data is updated to 2017. This Table replicates from Table 4 of the paper while using the alternative DV specification discussed in the supplemental information. In this model specification, states remain in the dataset through all mandate revisions and are only removed once they have finished revising their ASD mandates.

Table J. Replication of Table 3 Interest Group Industry Measures with Generalized Interest Group Density

|  | Model 3: No Policy Need | Model 4: With Policy Need |
| --- | --- | --- |
| VARIABLES | DV: Mandate Generosity | DV: Mandate Generosity |
|  |  |  |
| Dem. Govt. Control | 0.37** | 0.30* |
|  | (0.161) | (0.176) |
| Citizen Ideology | 0.04*** | 0.04** |
|  | (0.015) | (0.016) |
| Median Household Income | -0.00005 | -0.00005 |
|  | (0.00004) | (0.00004) |
| Percent Uninsured | 0.17* | 0.14 |
|  | (0.092) | (0.102) |
| Percent Employer Sponsored | 0.15** | 0.12* |
|  | (0.063) | (0.063) |
| Policy Diffusion | -0.71 | -0.85 |
|  | (0.971) | (0.971) |
| Policy Need – # with ASD |  | -43.32 |
|  |  | (87.810) |
| Percent Self Insured | 0.03 | 0.03 |
|  | (0.038) | (0.039) |
| Interest Group Density | 0.00** | 0.00** |
|  | (0.000) | (0.000) |
| Legislative Professionalism | -0.72 | -0.38 |
|  | (1.682) | (1.718) |
| T | -0.72 | 4.21 |
|  | (0.523) | (3.532) |
| T2 | 0.13** | -0.28 |
|  | (0.062) | (0.302) |
| T3 | -0.004* | 0.01 |
|  | (0.002) | (0.008) |
|  |  |  |
| Observations | 583 | 335 |
| Pseudo R-Squared | 0.19 | 0.14 |
| Log Pseudolikelihood | -194.44 | -176.54 |

Robust standard errors in parentheses

*** p<0.01, ** p<0.05, * p<0.10

NOTES: Results obtained using ordinal logistic regression including cubic polynomials for time and results clustered by state. Where no mandate was enacted, data is updated to 2017. This Table replicates from Table 3 of the paper while replacing our measures of health interest group and insurance interest group presence with a more generalized overall interest group density measure. This measure was developed by Lowery et al. (2015) and is available through the Michigan State Correlates of State Policy Dataset.

Table K. Replication of Table 3 – Model 2 Interacting Interest Group and Policy Need Measures

|  | Model 4: With Policy Need |
| --- | --- |
| VARIABLES | DV: Mandate Generosity |
|  |  |
| Dem. Govt. Control | 0.30* |
|  | (0.157) |
| Citizen Ideology | 0.04** |
|  | (0.016) |
| Median Household Income | -0.00005 |
|  | (0.00003) |
| Percent Uninsured | 0.15 |
|  | (0.105) |
| Percent Employer Sponsored | 0.12* |
|  | (0.061) |
| Policy Diffusion | -0.94 |
|  | (0.941) |
| Policy Need – # with ASD | 88.44 |
|  | (283.629) |
| Percent Self Insured | 0.03 |
|  | (0.037) |
| Interest Groups - Health | 0.03 |
|  | (0.025) |
| Interest Groups - Insurance | -0.05 |
|  | (0.059) |
| Insurance Int. Group*Policy Need | 12.36 |
|  | (17.801) |
| Health Int. Group*Policy Need | -5.14 |
|  | (6.458) |
| Legislative Professionalism | -0.99 |
|  | (1.943) |
| T | 3.70 |
|  | (3.998) |
| T2 | -0.22 |
|  | (0.335) |
| T3 | 0.01 |
|  | (0.009) |
|  |  |
| Observations | 335 |
| Pseudo R-Squared | 0.15 |
| Log Pseudolikelihood | -174.07 |

Robust standard errors in parentheses; *** p<0.01, ** p<0.05, * p<0.10

NOTES: Results obtained using ordinal logistic regression including cubic polynomials for time and results clustered by state. Where no mandate was enacted, data is updated to 2017. This Table replicates from Table 3 of the paper while adding interactions between the two interest group measures and the number of children in each state with ASD (policy need).

Table L. Continuous Alternative DVs – No Policy Need

|  | Model 1 | Model 2 | Model 3 | Model 4 |
| --- | --- | --- | --- | --- |
| VARIABLES | Continuous Expenditure Cap – 100K Cap | Continuous Expenditure Cap – No Cap Missing | Continuous Age Cap – 100 Yr. Cap | Continuous Age Cap – No Age Cap Missing |
|  |  |  |  |  |
| Dem. Govt. Control | 603.36 | 306.37 | 0.78** | 0.27* |
|  | (551.338) | (303.847) | (0.320) | (0.142) |
| Citizen Ideology | 128.15*** | 65.36** | 0.01 | 0.03*** |
|  | (36.540) | (26.470) | (0.020) | (0.010) |
| Median HH Income | -0.03 | -0.09 | -0.00008 | -0.00002 |
|  | (0.084) | (0.061) | (0.00005) | (0.00002) |
| Per. Uninsured | 435.50 | 196.43 | -0.05 | 0.14** |
|  | (276.847) | (148.597) | (0.182) | (0.064) |
| Per. Empl. Sponsored | 228.48 | 132.26 | 0.11 | 0.07 |
|  | (150.134) | (105.747) | (0.078) | (0.044) |
| Policy Diffusion | -3,057.34 | 3,359.83 | -1.31 | 0.27 |
|  | (5,467.811) | (3,637.652) | (3.362) | (1.649) |
| Per. Self Insured | 165.26 | 35.04 | 0.04 | 0.03 |
|  | (117.848) | (80.943) | (0.087) | (0.032) |
| Int. Groups - Health | 38.24* | 24.11** | 0.02** | 0.01** |
|  | (21.829) | (11.557) | (0.009) | (0.005) |
| Int. Groups - Insurance | -45.65 | -15.94 | -0.02 | -0.02 |
|  | (41.473) | (34.886) | (0.021) | (0.011) |
| Legislative Prof. | -3,328.05 | -4,730.76 | 7.84** | -1.26 |
|  | (5,918.855) | (4,134.128) | (3.832) | (2.551) |
| T | -3,191.51*** | -1,456.16 | -1.55*** | -0.65* |
|  | (1,093.125) | (901.974) | (0.572) | (0.339) |
| T2 | 443.85** | 214.68 | 0.26** | 0.10* |
|  | (182.954) | (150.316) | (0.105) | (0.056) |
| T3 | -11.92 | -5.95 | -0.01** | -0.002 |
|  | (8.161) | (6.946) | (0.005) | (0.003) |
| Constant | -26,860.16** | -9,457.42 | -4.90 | -7.68** |
|  | (13,086.151) | (7,424.396) | (5.583) | (3.458) |
|  |  |  |  |  |
| Observations | 582 | 510 | 583 | 538 |
| R-squared | 0.11 | 0.15 | 0.08 | 0.14 |

Robust standard errors in parentheses; *** p<0.01, ** p<0.05, * p<0.10

Notes: Model 1 presents an ordinary least squares regression where the DV represents a continuous insurance mandate expenditure cap. For states with a mandate but no expenditure cap, this value is set arbitrarily above all other states at $100,000. Model 2 replicates Model 1 but codes all states with a mandate but no expenditure cap as missing values for all state-years. Model 3 presents an ordinary least squares regression where the DV represents a continuous insurance mandate age cap. For states with a mandate but no age cap, this value is set arbitrarily above all other states at 100 years. Model 4 replicates Model 3 but codes all states with a mandate but no age cap as missing values for all state-years. All models have results clustered by states and include cubic polynomials for time.

Table M. Continuous Alternative DVs – With Policy Need

|  | Model 1 | Model 2 | Model 3 | Model 4 |
| --- | --- | --- | --- | --- |
| VARIABLES | Continuous Expenditure Cap – 100K Cap | Continuous Expenditure Cap – No Cap Missing | Continuous Age Cap – 100 Yr. Cap | Continuous Age Cap – No Age Cap Missing |
|  |  |  |  |  |
| Dem. Govt. Control | 746.23 | 562.68 | 1.17* | 0.46* |
|  | (972.820) | (558.708) | (0.585) | (0.242) |
| Citizen Ideology | 195.99*** | 93.68* | 0.02 | 0.05*** |
|  | (69.151) | (50.561) | (0.038) | (0.018) |
| Median HH Income | -0.12 | -0.16 | -0.0001 | -0.00006 |
|  | (0.176) | (0.137) | (0.0001) | (0.00006) |
| Per. Uninsured | 707.07 | 276.90 | 0.04 | 0.24 |
|  | (617.187) | (412.159) | (0.364) | (0.151) |
| Per. Empl. Sponsored | 330.19 | 231.76 | 0.25 | 0.20* |
|  | (348.846) | (278.658) | (0.193) | (0.110) |
| Policy Diffusion | -6,711.57 | 1,748.04 | -3.67 | -0.91 |
|  | (6,452.994) | (4,147.217) | (3.878) | (1.822) |
| Per. Self Insured | 795,135.35 | 163,562.19 | -254.83 | -324.08* |
|  | (715,780.544) | (538,353.053) | (403.090) | (182.357) |
| Int. Groups - Health | 189.41 | 87.97 | 0.04 | 0.05 |
|  | (194.124) | (158.175) | (0.145) | (0.056) |
| Int. Groups - Insurance | 37.10 | 33.66* | 0.02 | 0.02** |
|  | (35.659) | (18.862) | (0.016) | (0.009) |
| Legislative Prof. | -23.66 | -17.70 | -0.02 | -0.02 |
|  | (74.331) | (56.121) | (0.039) | (0.021) |
| T | 364.47 | -6,727.94 | 16.87** | -1.67 |
|  | (9,782.875) | (6,717.848) | (6.720) | (3.605) |
| T2 | -14,932.03 | -1,741.52 | -7.09 | -3.12 |
|  | (13,153.394) | (8,764.464) | (7.512) | (3.621) |
| T3 | 1,653.25 | 265.15 | 0.90 | 0.38 |
| Dem. Govt. Control | (1,302.883) | (874.622) | (0.727) | (0.357) |
|  | -49.30 | -7.05 | -0.03 | -0.01 |
|  | (39.682) | (27.708) | (0.022) | (0.011) |
| Constant | -5,434.07 | -18,214.01 | -1.28 | -9.59 |
|  | (41,605.789) | (33,512.785) | (27.235) | (12.636) |
|  |  |  |  |  |
| Observations | 334 | 299 | 335 | 312 |
| R-squared | 0.13 | 0.13 | 0.12 | 0.14 |

Robust standard errors in parentheses

*** p<0.01, ** p<0.05, * p<0.10

Notes: Table M replicates Table L while adding a measure of policy need to each model specification.
